# Traditional Chinese Herbal Patch for Short-Term Management of Knee Osteoarthritis: A Randomized, Double-Blind, Placebo-Controlled Trial

**DOI:** 10.1155/2012/171706

**Published:** 2012-02-12

**Authors:** Xuezong Wang, Yuelong Cao, Jian Pang, Jiong Du, Chaoqing Guo, Ting Liu, Songpu Wei, Yuxin Zheng, Rongming Chen, Hongsheng Zhan

**Affiliations:** ^1^Shi's Center of Orthopedics and Traumatology, Shuguang Hospital, Shanghai University of Traditional Chinese Medicine, 528 Zhangheng Road, Pudong New Area, Shanghai 201203, China; ^2^Institute of Traumatology & Orthopedics, Shanghai Academy of Traditional Chinese Medicine, Shanghai 201203, China; ^3^Teaching and Research of Surgery, Nanjing University of Chinese Medicine, 138 Xianlin Avenue, Jiangsu 210046, Nanjing, China

## Abstract

*Objective*. To assess the short-term efficacy and safety of two kinds of Traditional Chinese herbal patches, Fufang Nanxing Zhitong Gao (FNZG) and Shangshi Jietong Gao (SJG), for painful knee osteoarthritis (OA). *Methods*. Patients were randomly enrolled in a double-blind, placebo-controlled study to receive FNZG (*n* = 60), SJG (*n* = 60), or placebo patch (*n* = 30) for 7 days. Outcome measures included visual analogue scale (VAS), Western Ontario and McMaster Universities Osteoarthritis Index (WOMAC), and Traditional Chinese Medicine Syndrome Questionnaire (TCMSQ) subscale. *Results*. Although there was no significant difference among, three groups in short-term pain management, patients receiving FNZG got significant improvement in symptom of fear of coldness as compared with placebo patch (*P* = 0.029). The most common local adverse events of rash, itching, erythema, and slightly damaged skin were observed in 7% of participants. *Conclusions*. FNZG may be a useful treatment for symptom of knee OA and merits long-term study in broader populations.

## 1. Introduction

Osteoarthritis (OA) of the knee increases in prevalence with age and is a major cause of pain, locomotor disability worldwide [1, 2]. OA is characterized by a series of pathological changes in the whole joint, including articular cartilage degeneration and destruction, subchondral sclerosis, synovial hyperplasia, joint capsule contracture, ligament laxity or contracture, and muscles weakness and atrophy [1, 3]. The main factors consistently associated with knee OA were obesity, prior knee injuries, female, gender and older age [1, 3]. Until now, no ideal disease modifying medications exist in short-term pain management of knee OA.

Patients with knee OA usually call for a better pain control and less adverse events (AEs) within a short-term period. Therefore, as aging and comorbidities increase, a more convenient approach is eagerly to be applied. In China and increasingly worldwide, among pharmacological therapies, the recent guidelines recommended topical medications as an alternative or adjunctive therapy, or even as first-line therapy for knee OA [2–4]. Before oral administration, topical treatments are usually recommended to relieve mild or moderate pain of knee OA, because its safety profile is fairly well [3].

A great number of Chinese doctors have prescribed some forms of Traditional Chinese Medicine (TCM) to patients with knee OA, of which the herbal patch is a common approach and has long been a standard treatment [3, 4]. The patch, called “Bo Tie” in ancient times, is a unique topical formulation of TCM, which is made through frying edible vegetable oil and the herbs, discarding the residue, then mixing with *Componere Hydrargyrum* (Shenyao) and cooling, and coating with cloth or paper finally. When affixed to the injured area or acupoint, it could alleviate symptoms in a certain part of body, such as bruises or muscle pain [5].

The herbal patch is used under the principle of syndrome differentiation based on TCM theory. OA is known as the bone obstruction disease, which means either the limbs or the joints suffer from pain, stiffness, and/or malfunction due to invasions of wind cold or dampness into knees accompanied by the disharmony of Qi and blood, consequently leading to the syndrome of “cold dampness” and “blood stasis” [6]. Accordingly, different herbal formulations were prepared for different symptoms due to cold evil and dampness evil.

Currently, Fufang Nanxing Zhitong Gao (FNZG) and Shangshi Jietong Gao (SJG) are two most prescribed herbal patches in China with the indication for the management of painful knee OA. Published literatures documented that FNZG or SJG had a better efficacy for knee OA or joint pain [7–13]. However, these interventions have, to date, not been rigorously evaluated, and such methods have been regarded mostly effective in case reports. Nevertheless, only one article about randomized controlled trial of FNZG for knee OA was published, but interpretation of its results was limited by methodological quality, including restricted demographics characteristics, absence of standardized outcome measures, and poor statistical analysis [11]. In addition, some AEs also have been reported in the literatures [14–17]. Most critically, although Traditional Chinese herbal patch has been applied over centuries, there were no placebo-controlled trials published for validating its effectiveness in treating knee OA.

Therefore, we conducted a randomized, double-blind, and placebo-controlled trial to explore the effectiveness and safety of FNZG and SJG, for the management of painful knee OA in a short-term design. We hypothesized that at the end of the 7-day treatment, patients receiving FNZG or SJG would have greater reduction in pain and better improvements in symptoms than those in the placebo control group.

## 2. Materials and Methods

### 2.1. Study Design

This was a prospective 7-day, 3-arm, and double-blind study in subjects with painful knee OA which compared FNZG versus the placebo, and SJG versus the placebo with imbalanced design, the allocation ratio was 2: 2 : 1 for FNZG, SJG, and placebo, respectively. The study was approved by the Ethical Committee of South Hospital, South Medical University and registered in Clinical Trials of Chinese Cochrane Center (Registered no. ChiCTR-TRC-10000963).

### 2.2. Setting and Participants

The study was conducted in the outpatient of Shuguang Hospital, a teaching hospital affiliated to Shanghai University of TCM.

Patients with painful knee OA were recruited from the previous study: discontinue interval [6], clinic patient database, and the community by batch. Baseline questionnaires were not filled out until at least 15 participants had accumulated, and then signed informed consent if eligible.

The eligibility criteria consisted of knee OA diagnostic criteria of Chinese Orthopaedic Association (COA) [3] and TCM syndrome [18], male or female between 40 and 70 years of age, evidence of idiopathic OA of at least one knee, patient assessment of OA pain in index knee above 20 mm on the 100 mm visual analogue scale (VAS) at baseline. Exclusion criteria included (1) pain greater than 20 mm on VAS in the nonindex knee (either at rest or with movement); (2) prior injection or arthroscopy of study knee within 3 months; (3) an injury or surgery to the same body region within the prior 6 months or a lifetime history of 3 or more injuries/surgeries to the injured body region; (4) signs of clinically important active inflammation of the study knee joint including redness, warmth, and/or a large, bulging effusion with the loss of normal contour at the screening and/or baseline visits; (5) crystalline-induced synovitis (e.g., gout, pseudogout, nonsteroid arthritis, hydroxyapatite deposit), rheumatoid arthritis, psoriatic arthritis, septic arthritis, fibromyalgia, systemic lupus, erythematosus collagen vascular disease; (6) with severe cardiovascular, lung, liver, kidney, and hematopoietic system and other serious primary diseases (such as myocardial infarction, heart failure), cancer, and neurological joint pain; (7) complications of the disease affected joints, such as psoriasis, brown yellow disease, metabolic bone disease, acute trauma; (8) using nonsteroidal anti-inflammatory drugs (NSAIDs) and received glucocorticoid treatment within 2 and 4 weeks, respectively; (9) having experienced a history of skin irritation using patches; (10) other reasons in the opinion of investigators that the subject did not suit, for example, participating in other trials.

OA at other sites besides the knee was permissible. In case of poor quality, repeated images were acquired.

### 2.3. Interventions

Participants in each arm received FNZG, SJG, or placebo patch, respectively. All patches had the same size of 10 cm × 13 cm and were matched with each other for taste, color, and package. One kind of patch in 7 days was packed into a box and labeled patch code in eye-catching position. Meanwhile, patches were stored, administrated, and dispensed by a pharmacist in a larger container at the scientific research pharmacy.

The FNZG (China State Food and Drug Administration (SFDA) approval no. Z10970019, Jiangsu Nanxing Pharmaceutical Co., Ltd.) contained active ingredients of Hypaconitine (C_33_H_45_NO_10_) and Eugenol (C_10_H_12_O_2_), which were extracted mainly from raw *Rhizoma Arisaematis* (Sheng Tiannanxing), raw *Radix Aconiti* (Sheng Chuanwu), and *Flos Caryophylli* (Dingxiang). In addition, it consisted of other 9 kinds of herbs: *Cortex Cinnamomi* (Rougui),* Radix Angelicae Dahuricae* (Baizhi), *Herba Asari* (Xixin),* Rhizoma Chuanxiong* (Chuanxiong), *Radix Cynanchi Panicuclati* (Xuchangqing), processed *Olibanum* (Zhi Ruxiang), processed *Myrrha* (Zhi Moyao), *Camphora* (Zhangnao), and *Borneolum Syntheticum* (Bingpian).

The SJG (SFDA approval no. Z32021099, Changzhou Shenghui Pharmaceutical Co., Ltd.) contained similar active ingredients with FNZG. It consisted of 17 kinds of herbs. Some were same as FNZG, such as raw *Rhizoma Arisaematis* (Sheng Tiannanxing), raw *Radix Aconiti* (Sheng Chuanwu),* Radix Angelicae Dahuricae* (Baizhi), *Cortex Cinnamomi* (Rougui), *Camphora* (Zhangnao), *Borneolum Syntheticum* (Bingpian), but* Radix Angelicae Pubescentis* (Duhuo), *Cortex Acanthopanacis* (Wujiapi),* Rhizoma Curcuma Longae* (Jianghuang), *Flos Carthami *(Honghua), *Folium Artemisiae Argyi *(Aiye), *Rhizoma Atractylodis *(Cangzhu), *Rhizoma Pinellia* (Banxia),* Semen Sinapis *(Baijiezi), *Semen Vaccariae *(Wangbuliuxing), raw *Radix Aconiti Kusnezoffii* (Sheng Caowu), and *Herba Menthae *(Bohe).

The placebo patch (approval no. 1640087, Zhejiang, Tongxiang, Hongshi Pharmaceutical Co., Ltd.) was acrylic pressure-sensitive adhesive tape.

According to the order of participants' attendance and patch code, a box of patch was dispensed from small to large numbers, which remained unchanged throughout the entire trial. Participants were required to cover the left or right index knee each day at bed time and remove 8 hours after application in the morning (not to exceed 12 hours) with a period of 7 days, which is similar with precious experimental and trial studies [9, 11, 19]. The VAS and time of patch application and removal were recorded daily in a diary for compliance purpose.

Participants were encouraged to maintain their regular activities and medications, including drugs of hypertension, diabetes and other conditions, but not attending physical therapy for knee OA other than using new analgesic. No rescue medication was provided throughout the trial, but patients have been fully informed to have the right to withdraw at any time when they feel unbearable symptoms of disease.

### 2.4. Outcome Measures

The primary efficacy measure used a VAS (score range 0–100 mm) walking on flat surface to assess the change from baseline to day 1 and day 7 in the patient's assessment of pain score. Secondary outcome measures were (1) patient global assessment of the change in Western Ontario and McMaster Universities Osteoarthritis Index (WOMAC, Likert scale version; score range 0–96) [20]. The total WOMAC score is a summation of the scores for each individual domain (pain (score range 0–20), stiffness (score range 0–8), and physical function (score range 0–68)); (2) WOMAC pain, stiffness, and physical function subscales; (3) subscale of investigator assessment knee OA condition measured by TCM Syndrome Questionnaire (TCMSQ; score range 0–24) (Table 1) [21]. Given the reason that Chinese herbal medicine is applied under the principle of syndrome differentiation according to TCM philosophy, TCMSQ is used as an endpoint in this trial with higher scores indicating more severe syndrome. Adherence and occurrence of AEs were also assessed.

### 2.5. Safety Assessments

All patients were screened clinically, biochemically, and by 12-lead electrocardiogram (ECG) for any sign of conditions on day 0 and day 7 and repeated if necessary. Vital signs were monitored, using a standard adverse-event case report form at each visit. This form included a description of all undesirable experiences, such as (1) symptoms of skin itching, rash, blisters and skin damage; (2) local pain and discomfort; (3) emergency situations such as fracture or joint floating body; (4) abnormality of kidney and liver functional tests. For all AEs linked to patches, actions taken would be recorded and followed until resolution of the event throughout the study.

### 2.6. Sample Size

We determined the sample size based on previous data that the efficacy of FNZG, SJG, and placebo patch was 59.6%, 33.1%, and 10%, respectively [11, 13]. According to the 2 : 2 : 1 ratio, we estimated that FNZG (*n* = 52), SJG (*n* = 52), and placebo patch (*n* = 26) were sufficient to give 0.8 power at 0.05 alpha level. Allowing for dropout, 150 participants were included.

### 2.7. Randomization and Blinding

Participants were randomly and blindly assigned to FNZG group (*n* = 60), SJG group (*n* = 60), or placebo group (*n* = 30) by the Evidence-Based Medicine Center of Beijing University of Chinese Medicine. The investigators, the subjects, those evaluating outcomes, data entry personnel, and statistician were blinded to treatment allocations.

### 2.8. Data Analysis

Data was managed through the online facility established by the Evidence-Based Medicine Center of Beijing University of Chinese Medicine (http://202.204.46.209/cw/) and was analyzed on the full analysis set (FAS) with the intent-to-treat principle, the per-protocol set (PPS) for adherence, and safety set (SS) for adverse events. Data for participants who lost to followup was estimated using the last-observation-carried-forward (LOCF) method for the primary outcome measure. We compared three group changes from pre- and postscores with one-factor analysis of variance (ANOVA). We evaluated for potential effects of confounding or interaction with treatment by covariates, including age, sex, body mass index (BMI), disease duration, and knee pain. Results are shown with 95% confidence interval (95% CI). All the statistical tests were used two sided and set at the 5% level. Statistical analysis was performed using SPSS version 16.0.

## 3. Results

### 3.1. Participants

**Figure 1:**
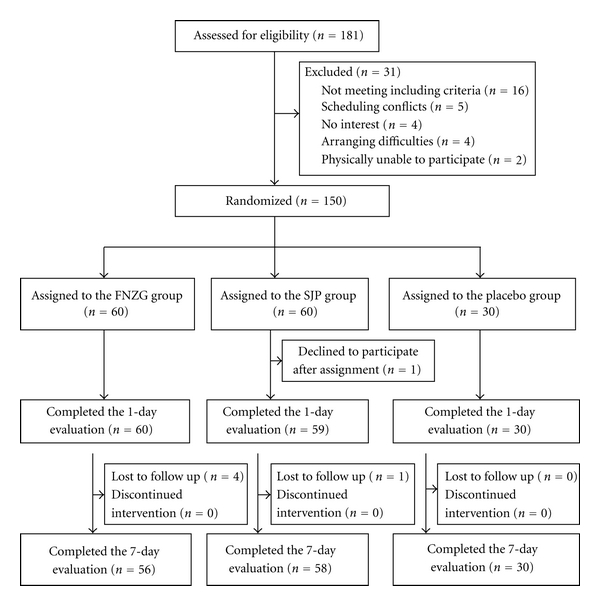
Flow diagram of the trial progress about enrollment, randomization, intervention, and completion of 1-day and 7-day evaluations.

Between June 2010 and January 2011, we screened 181 patients with painful knee OA by telephone and interview. 31 patients were excluded for various reasons, and 150 qualified participants were randomly assigned to FNZG group, SJG group, or placebo group, but one person declined to participate after assignment, the specific reason was unknown (Figure 1).

### 3.2. Baseline Characteristics of the Patients

The race of subjects was Han Chinese, and the majority of them had greater than or equal to junior school education.  Table 2 summarizes the main baseline characteristics of the 149 patients based on FAS. Participants had a mean age of 59.3 years, and 91% (136) were women. On average, participants had 4.4 years of knee pain and BMI of 23.5 kg/m^2^. At baseline, the demographic and clinical characteristics were reasonably well balanced among the 3 groups except duration of knee pain (*P* = 0.046), and there was also somewhat difference about greater knee OA severity in the placebo group (Table 2).

### 3.3. Primary Outcome Measure

**Figure 2:**
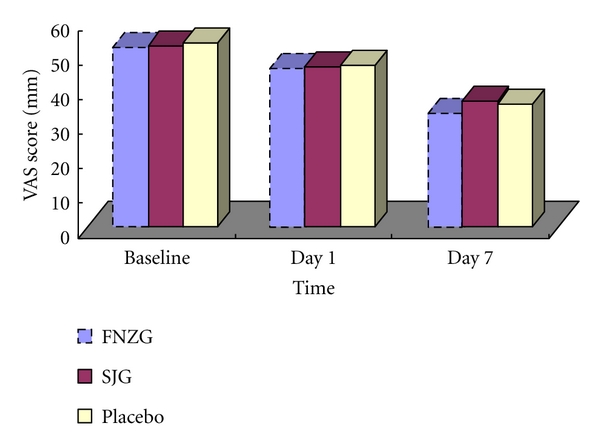
Mean changes of VAS score by treatment group during the 7-day intervention period.

All three groups improved with respect to a decrease from baseline to day 1 and day 7 in the VAS score. Similar improvements from baseline were observed at day 1. Improvements from baseline were the greatest in FNZG group and the smallest in SJG group at day 7. However, the difference between placebo (−17.68 points, [95% CI, −22.16 to −13.21]) and FNZG (−19.02 points, [95% CI, −23.23 to −14.81]; *P* = 0.858) as well as difference between placebo and SJG (−16.04 points, [95% CI, −19.30 to −12.79]; *P* = 0.789) were not statistically significant (Table 3; Figure 2).

### 3.4. Secondary Outcome Measures

Improvements from baseline to day 7 in the 3 WOMAC domains and total score were seen in three groups. As compared with the changes of placebo and FNZG group, SJG group had greater improvements in the scores for 3 WOMAC domains and total, but no significant differences were documented (Table 4).

Notably, FNZG group had a significantly greater decrease in the item of fear of coldness as assessed by TCMSQ than that in placebo group (−0.89 points, [95% CI, −1.23 to −0.56] versus −0.29 points, [95% CI, −0.56 to −0.01]; *P* = 0.029), but SJG group did not reach a significant reduction (−0.69 points, [95% CI, −0.96 to −0.42]; *P* = 0.172).

All treatment effects remained unchanged after adjusting for duration of knee pain, and no interactions with treatment were noted.

### 3.5. Adherence and Adverse Events

The rate of attendance was 93%, 97%, and 100%, respectively, for FNZG, SJG and placebo at the 7-day followup (Table 5). 1 person withdrew from the study by 1 day and 6 persons by 7 days. 5 patients in FNZG and 3 in SJG group missed some outcomes visits but completed other followup evaluations.

The most common AEs of FNZG and SJG were rash, itching, slightly damaged skin, or erythema in 7% of participants based on SS. These symptoms disappeared within 1 day to 3 days after discontinuation. One participant in the FNZG group reported an increase in knee pain and swelling (urticaria) after three-day usage. This was resolved by discontinuation and giving anther kind of herbal patch (Morengao) and taking Loxoprofen sodium tablet 60 mg immediately (Daiichi Sankyo Pharmaceutical (Shanghai) Co., Ltd.). No server AEs were reported during the study.

## 4. Discussion

Our randomized, double-blind, placebo-controlled study revealed that Traditional Chinese herbal patch FNZG might be a useful therapy for knee OA in improving symptom of fear of coldness as measured by TCMSQ at 7 days. No serious AEs were reported in the study participants, indicating that it may be a safe treatment for knee OA.

Several studies lacking placebo control have evaluated the efficacy and safety of FNZG or SJG in the management of knee pain [7–12] and TCM syndrome [13, 17]. One also showed that FNZG was effective as diclofenac cream [22]. Furthermore, AEs of FNZG or SJG including rash, itching, slightly damaged skin, or erythema were also reported in previous trials [8, 9, 11, 17, 22], but no allergic purpura of FNZG was found [16]. Withdrawal of FNZG participants was smaller than our trial (2/85 versus 4/60) [9].

According to features of knee OA, we chose two widely applied patches, FNZG and SJG, to rigorously evaluate their efficacy and safety. Our outcome measures capture both knee OA symptoms and the whole body situation. VAS is a simple and frequently used method for the assessment of variations in intensity of pain. In clinical practice, the pain relief is often considered as a measure of the efficacy of treatment [23]. WOMAC is a widely used outcome measure for OA treatments and is a validated scale to assess OA pain, stiffness, and physical function [24, 25]. WOMAC is detailed in the description of local symptoms of OA, whereas TCMSQ includes whole body's assessment in different phases of OA. TCM syndrome is different from the disease name; it may not only include local symptoms which are part of the disease but also symptoms from the whole body situation [6]. Therefore, TCMSQ could provide complementary information on outcome measures and represent clinically meaningful effects that were usually neglected by conventional scales. Given this consideration, swelling, range of motion, muscles fatigue, and fear of coldness, as alternative endpoints, could be concerned in the future study if available.

Although biological mechanism of Traditional Chinese herbal patches in improving the clinical consequence of knee OA is unclear, its analgesic and improvement of cold-dampness evil components likely play an important role. First, herbal patches may act as the kneepad. It has been shown to have slight fixation effect and could help patients overcome fear of pain as tape [26, 27]. Second, since TCM believes that disease is linked with the aging and feebleness, long-term strain, invasion of wind, cold, or dampness evil [12], herbal patches could treat local and even systemic conditions through the way of dispelling cold evil, removing dampness evil, activating the blood, and resolving stasis [28]. Third, it has a significant analgesic and anti-inflammatory effect on local microcirculation [29]. Some experimental data suggested that blood levels of prostaglandin E2 (PGE2), interleukin-1 (IL-1) and interleukin-6 (IL-6), were decreased, while *β*-endorphin (*β*-EP) was increased in patients with knee OA after using FNZG [30, 31]. Furthermore, it may have a potential influence on neurochemical and immune system, thus improving TCM syndrome in the whole body situation.

Our study had some limitations. The biggest limitation was the relatively short-term. It should be noted that our primary aim was to assess the effectiveness and safety of FNZG and SJG for painful knee OA for 7 days, and we determined the similar intervention period on published data [9, 11, 19]. Although OA is a chronic and degenerative disease, extension of pain always has fluctuation over the course; that is, one-week period could be in line with acute OA-pain flare episodes. This treatment period is also consistent with clinical practice, as patients' adherence would be extremely poor if pain could not be satisfactorily controlled by topical treatment alone within a short period. Since we only recruited patients with pain intensity of 20 mm or more on VAS, participants may have equal and higher responding to treatment than those without such restriction. Similarly, many OA trials have set “flare” group in which participants must have sensitive respond to treatment, such as symptoms getting back easily while stopping medication [32]. Future studies should conduct long-term followup to verify whether the benefits are maintained. Another limitation was the smaller response rate to detect the desired significance level. It was not due to sample size alone which was rigorously calculated, rather, it could be explained by the fact that the cohort we recruited with relatively mild degree of knee pain at baseline (VAS = 52.3 mm) might be insensitive in identifying improvement compared with high-response populations. We had attempted to analyze data by stratification of knee pain, but the result still unchanged, indicating a really no difference in this time interval. In addition, although VAS is a validated measure for pain, its estimate performed by patients with chronic pain may be imprecision [23, 33]. Therefore, both objective and subjective status of knee pain assessed separately by participant and investigator could be considered in future work.

## 5. Conclusion

Our preliminary findings indicate that Traditional Chinese herbal patches FNZG or SJG may have limited effect to improve knee pain, stiffness, and physical function of knee OA in a short-term treatment. The local adverse events of rash, itching, erythema, and slightly damaged skin were reported in few people. However, patients receiving FNZG could get significant improvement in symptom of fear of coldness as measured by TCMSQ.

## Figures and Tables

**Table 1:** Subscale of investigator assessment of knee OA condition measured by Traditional Chinese Medicine Syndrome Questionnaire (TCMSQ).

Items	Assessment criteria			
	0 point	2 points	4 points	6 points
Swelling	None	Suspected floating patella test	Positive floating patella test	Significantly
Range of motion	Move freely	120°~140°	100°~120°	<100°
Fatigue in the region of waist and knee muscles	None	Occasionally, but not affected the life and work	Occurred only after working	Persisted during the life
Fear of coldness	None	Not palpable over knee joints when palpation	Palpable over knee joints when palpation	Obviously, needed clothing to protect the whole body

**Table 2:** Baseline characteristics of the study participants.

Variable	Placebo (*n* = 30)	FNZG (*n* = 60)	SIG (*n* = 59)	Total (*n* = 149)
Demographics				

Han Chinese, no.(%)	30 (100)	60 (100)	59 (100)	149 (100)
Women, no.(%)	27 (90)	53 (88)	56 (95)	136 (91)
Age, years	60.4 ± 8.0	58.5 ± 7.7	59.6 ± 6.1	59.3 ± 7.1
Body mass index, kg/m^2^	23.4 ± 2.7	23.5 ± 3.1	23.7 ± 2.7	23.5 ± 2.9

Disease condition				

Duration of knee pain (on study knee), years*	4.6 ± 3.0	5.1 ± 4.1	3.5 ± 3.0	4.4 ± 3.5
Patient VAS (study knee; range 0–100 mm)	53.3 ± 15.2	51.7 ± 15.0	52.5 ± 14.7	52.3 ± 14.8
WOMAC pain (range 0–20)	10.6 ± 4.6	9.2 ± 4.3	9.3 ± 3.6	9.5 ± 4.1
WOMAC stiffness (range 0–8)	3.6 ± 1.9	3.3 ± 1.9	3.4 ± 1.9	3.4 ± 1.9
WOMAC physical function (range 0–68)	28.3 ± 16.0	23.9 ± 14.6	25.9 ± 13.8	25.5 ± 14.6
WOMAC total score (range 0–96)	42.5 ± 22.0	36.3 ± 20.1	38.5 ± 18.2	38.4 ± 19.8

Investigator TCMSQ subscale				

Swelling (range 0–6)	2.7 ± 1.7	2.47 ± 1.1	2.14 ± 1.4	2.4 ± 1.3
Range of motion (range 0–6)	2.8 ± 1.1	2.87 ± 1.1	2.78 ± 1.3	2.8 ± 1.2
Fatigue in the region of waist and knee muscles (range 0–6)	3.5 ± 1.5	3.40 ± 1.2	3.22 ± 1.3	3.4 ± 1.3
Fear of coldness (range 0–6)	3.9 ± 1.3	3.92 ± 1.4	3.83 ± 1.4	3.9 ± 1.4

Self-reported comorbidities, no. (%)				

Heart disease	0 (0)	3 (5)	1 (2)	4 (3)
Hypertension	6 (20)	9 (15)	9 (15)	24 (16)
Diabetes	1 (3)	3 (5)	2 (3)	6 (4)

Values are the mean ±SD or the number (percentage). *P* values were calculated by one-factor analysis of variance (ANOVA). VAS: visual analog scale; WOMAC: Western Ontario and McMaster Universities Osteoarthritis Index; TCMSQ: Traditional Chinese Medicine Syndrome Questionnaire. *P = 0.046.

**Table 3:** Changes in primary outcome.

Variable	Placebo (*n* = 30)	FNZG (*n* = 60)	SIG (*n* = 59)
Patient VAS (range 0–100 mm)			

Day 1	46.73 (40.97, 52.49)	45.72 (41.50, 49.94)	46.39 (42.73, 50.05)
Improvement from baseline	−6.58 (−9.41, −3.76)	−6.05 (−8.07, − 4.03)	−6.09 (−7.60, − 4.58)
*P* value		0.909	0.919

Day 7	35.63 (28.94, 42.33)	34.19 (29.58, 38.80)	36.71 (33.12, 40.29)
Improvement from baseline	−17.68 (−22.16, − 13.21)	−19.02 (−23.23, − 14.81)	−16.04 (−19.30, − 12.79)
*P *value		0.858	0.789

Values are the mean (95% confidence interval).* P* values were calculated by one-factor analysis of variance (ANOVA). VAS: visual analog scale.

**Table 4:** Changes in secondary outcomes.

Variable	Placebo (*n* = 30)	FNZG (*n* = 60)	SIG (*n* = 59)
WOMAC: Pain (range 0–20)			

Day 7	8.59 (6.92, 10.26)	7.45 (6.34, 8.56)	6.79 (5.91, 7.68)
Improvement from baseline	−1.97 (−2.50, − 1.43)	−2.02 (−2.57, − 1.47)	−2.52 (−3.02, − 2.02)
*P *value		0.988	0.310

Stiffness (range 0–8)			

Day 7	2.97 (2.22, 3.72)	2.55 (2.06, 3.05)	2.60 (2.19, 3.02)
Improvement from baseline	−0.66 (−1.00, − 0.31)	−0.77 (−0.99, − 0.55)	-0.78 (−1.10, −0.45)
*P *value		0.827	0.804

Physical function (range 0–68)			

Day 7	22.38 (16.31, 28.45)	19.77 (16.03, 23.51)	19.41 (16.17, 22.66)
Improvement from baseline	−6.10 (−8.34, − 3.87)	−5.04 (−6.21, − 3.86)	−6.71 (−8.24, −5.14)
*P *value		0.564	0.820

Total score (score 0–96)			

Day 7	33.93 (25.58, 42.28)	29.71 (24.52, 34.93)	28.81 (24.48, 33.14)
Improvement from baseline	−8.72 (−11.46, − 5.99)	−7.87 (−9.50, − 6.26)	−10.00 (−12.08, − 7.92)
*P *value		0.800	0.616

Investigator TCMSQ subscaleSwelling (range 0–6)			

Day 7	2.21 (1.73,2.70)	1.71 (1.50,2.07)	1.62 (1.33,1.91)
Improvement from baseline	−0.50 (−0.84, − 0.16)	−0.71 (−1.01, − 0.42)	−0.51 (−0.84, − 0.20)
*P* value		0.587	0.996

Range of motion (range 0–6)			

Day 7	2.29 (1.78,2.79)	2.04 (1.74,2.33)	2.14 (1.84,2.43)
Improvement from baseline	−0.50 (−0.90, − 0.10)	−0.89 (−1.21, − 0.57)	−0.66 (−0.90, − 0.41)
*P* value		0.186	0.727

Fatigue in the region of waist and knee muscles (range 0–6)			

Day 7	3.00 (2.46,3.54)	2.89 (2.57,3.21)	2.55 (2.28,2.83)
Improvement from baseline	−0.57 (−0.99, − 0.16)	−0.46 (−0.79, − 0.14)	−0.66 (−0.94, − 0.37)
*P* value		0.870	0.917

Fear of coldness (range 0–6)			

Day 7	3.57 (3.04,4.10)	3.04 (2.63,3.44)	3.14 (2.75,3.52)
Improvement from baseline	−0.29 (−0.56, −0.01)	−0.89 (−1.23, −0.56)	−0.69 (−0.96, −0.42)
*P* value		0.029	0.172

Values are the mean (95% confidence interval). *P* values were calculated by one-factor analysis of variance (ANOVA). WOMAC: Western Ontario and McMaster Universities Osteoarthritis Index; TCMSQ: Traditional Chinese Medicine Syndrome Questionnaire.

**Table 5:** Summary of adherence and adverse events (AEs).

Variable	Placebo	FNZG	SIG	Total
Adherence no. (%)				
Day 1	30 (100)	60 (100)	59 (98)	149 (99)
Day 7	30 (100)	56 (93)	58 (97)	144 (96)
All AEs*				
Patch site reaction	0 (0)	4 (7)	4 (7)	8 (5)
Leading to study withdrawal	0 (0)	1 (2)	0 (0)	1 (1)
Resulting in hospitalization and death	0 (0)	0 (0)	0 (0)	0 (0)

Values are the number (percentage). *Based on 149 patients (60 FNZG, 59 SJG, 30 placebo).
